# 2092: Chronic branched-chain amino acid ingestion aggravates hilar neuron loss in a rodent model of temporal lobe epilepsy

**DOI:** 10.1017/cts.2017.200

**Published:** 2018-05-10

**Authors:** Shaun Evan Gruenbaum, Roni Dhaher, Amedeo Rapuano, Tore Eid

## Abstract

OBJECTIVES/SPECIFIC AIMS: We previously developed a translationally relevant model of temporal lobe epilepsy (TLE) in which glutamine synthetase is irreversibility inhibited by methionine sulfoximine (MSO), resulting in spontaneous seizures and dentate hilar neuron loss. The objective of this study was to determine the effects of chronic BCAA ingestion on neuronal viability in the dentate hilus in the MSO model of TLE. METHODS/STUDY POPULATION: Sixteen rats were randomly divided into 2 groups: 8 rats drank a 4% aqueous solution of all 3 BCAAs (BCAA group) ad libitum for 31 days, and the other 8 rats drank regular water (control group) for the same period. After 10 days of drinking, a microinfusion cannula (Alzet osmotic pump, model 2004) was surgically implanted in the right dentate gyrus to continuously infuse MSO at a rate of 0.625 g/hour for 28 days. After 31 days of drinking, rats were perfused transcardially with 0.9% NaCl followed by 4% paraformaldehyde in phosphate buffer. The brains were removed and fixed, sectioned on a Vibratome at 50-μm thickness, and were mounted on a gelatin-coated slides and stained with NeuN. Neuron counts in the hilar region were performed ipsilateral and contralateral to the infusion site using a stereological technique. RESULTS/ANTICIPATED RESULTS: Rats in the BCAA group had 37% fewer neurons in the ipsilateral dentate hilus than the control group (5.8×10^−4^±6.8×10^−5^ vs. 8.9×10^−4^±5.6×10^−5^ cells, respectively, *p*<0.01). Similarly, rats in the BCAA group had 39% fewer neurons in the contralateral dentate hilus than the control group (5.0×10^−4^±5.8×10^−5^ vs. 7.0×10^−4^±3.4×10^−5^ cells, respectively, *p*=0.01). DISCUSSION/SIGNIFICANCE OF IMPACT: This study demonstrates that chronic ingestion of BCAAs aggravates hilar neuronal loss in a translationally relevant rodent model of MTLE. This study gives important insight into how BCAAs may affect neuronal viability. Although the role of BCAAs on seizure activity is poorly understood, these results suggest that BCAAs may play an important role in neurochemical modulation and neurotoxicity.

